# A couple of the first cousins born with hypotonia and maternal polyhydramnios

**DOI:** 10.1002/ccr3.8503

**Published:** 2024-02-07

**Authors:** Mousa Ahmadpour‐kacho, Yadollah Zahed Pasha, Samira Pournajaf

**Affiliations:** ^1^ Non‐Communicable Pediatric Diseases Research Center, Department of Pediatrics Babol University of Medical Sciences Babol Iran; ^2^ Non‐Communicable Pediatric Diseases Research Center, Department of Pediatrics Clinical Research Development Unit of Rouhani Hospital Babol University of Medical Sciences Babol Iran

**Keywords:** congenital, dystrophy, hypotonia, neonates, polyhydramnios

## Abstract

Congenital myotonic dystrophy should be considered in hypotonic infants with polyhydramniotic mothers with a positive history of myotonia.

**Abstract:**

Congenital myotonic dystrophy (CDM) is a predominantly maternally inherited disease and results from increased numbers of cytosine, thymine, and guanine (CTG) repeats in the unstable DNA regions and presents as hypotonia in the neonatal period and myotonia in adulthood. This report aims to present two cases of CDM. A first‐cousin couple was born and hospitalized due to hypotonia at birth and a maternal history of polyhydramnios during this pregnancy. The first‐born baby girl was admitted to the NICU with tachypnea and hypotonia, clubfoot, and frog‐like posture. The pregnancy was complicated by polyhydramnios. Interestingly, her first cousin was born the next day with a similar picture and history. Myotonia was detected in their mothers. The concurrent presence of hypotonia and polyhydramnios as well as maternal myotonia in a first cousin should be considered CDM until proven otherwise and this was confirmed by the EMG‐ NCV test.

## 1 INTRODUCTION

Congenital myotonic dystrophy (CDM) is an autosomal dominant genetic disorder, inherited primarily from the mother. Paternal transmission is rare with a few reported cases.^1^, ^2^, ^3^, ^4^, ^5^, ^6^, ^7^ What is unique about CDM is the hypotonia rather than myotonia that is detected in adulthood. Its manifestations include polyhydramnios, hypotonia, respiratory distress, and feeding difficulties that appear before and at birth, and motor and developmental delay and cognitive deficits that may appear later.^8^ The incidence of this disease is 2.1/100,000 live births in Canada.^9^ This disorder is associated with an increase in the number of CTG repeats in unstable DNA regions.^10^ In this report, we present a first‐cousin couple born with hypotonia and a positive history of maternal polyhydramnios.

## 2 CASE HISTORY/EXAMINATION

The patients are a couple of first cousins born 1 day apart in a private hospital and then transferred to our center on the first day after birth due to tachypnea and severe hypotonia.

**FIGURE 1:**
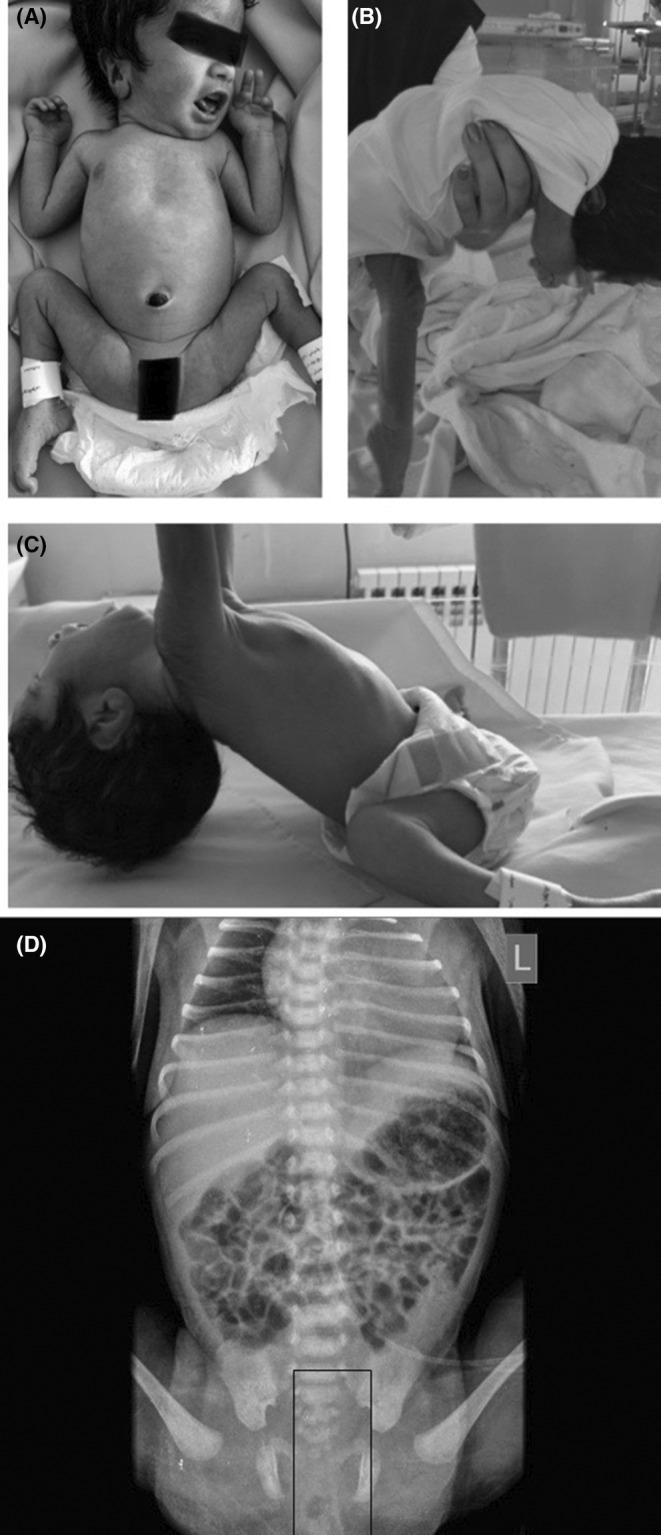
First neonate, frog‐like posture and club foot (A). Ventral suspension (B). Head lag in pull‐to‐sit maneuver (C). Right hemi‐diaphragm elevation (D).

The first neonate (N1) presented with significant hypotonia, tachypnea, and poor primitive reflexes including Moro and sucking. She was referred to our center due to these signs and symptoms. On general examination, a girl baby with hypotonia was manifested by decreased spontaneous movements and a frog‐like posture. On the other hand, she was alert by floppy. Parents were not relative to each other. On physical examination, we found a normal fontanel, hypertelorism, low set ears, high arc palate, poor sucking, difficulty in swallowing, and normal other cranial nerves (question about CN II, IX, X). No sign of birth injuries such as scalp swelling was present. Tongue fasciculation and light fixation were not detected. Tachypnea resolved in the first hours of life with supportive oxygen therapy. Heart and lungs auscultation was normal. Her abdomen was normal, with no tumors or enlarged organs. A normal spine, frog‐like legs, bilateral clubfoot, poor Moro reflex, and absent deep tendon reflex were present. Hypotonia was evident on the pull‐to‐sit maneuver (head lag), and vertical and ventral suspensions (Figure 1).

### 2.1 On maternal history

Her mother was a 28‐year‐old woman with a thin, bony face, atrophied temples and masseter muscles. She spoke loosely and had a positive history of limping and hand‐locking in early pregnancy. Her studies ended at school. Her previous pregnancy was uncomplicated and she gave birth to a normal son. She had polyhydramnios during this pregnancy with an AFI of 313 mm and complained of decreased fetal movements compared to her first pregnancy. This pregnancy was terminated by cesarean section at 38 weeks of gestation because of breech presentation.

**FIGURE 2:**
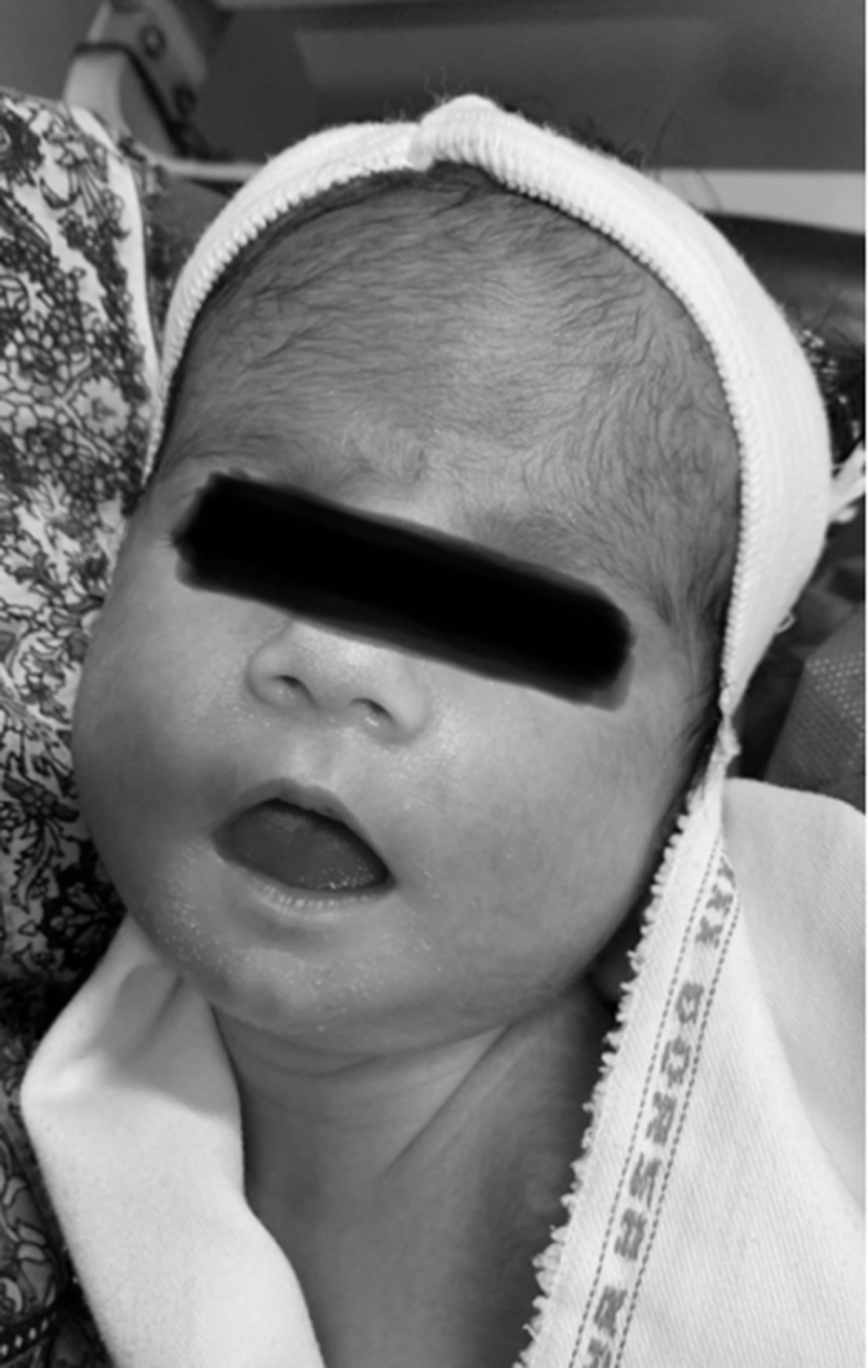
Typical tent‐shaped open mouth in neonate 2.

The second newborn (N2), (N1's cousin), was born 1 day after N1 at the same center and was transferred to our hospital because of hypotonia and poor sucking. In general inspection she looked alert and floppy with a tent‐like opened mouth (Figure 2). On physical examination, we found NL‐sized fontanel, high arc palate, a tent‐like opened mouth, poor sucking, no tongue fasciculation, NL spine, NL auscultation of heart and lungs, soft abdomen without organomegaly, NL female genitalia, hypotonic extremities, poor Moro reflex and reduced deep tendon reflex. Hypotonia was detected during horizontal suspension and head lag during pull‐to‐sit maneuver. Her symptoms were milder than N1.

### 2.2 Obstetric history

This was the first pregnancy and terminated at 39 weeks of gestation by cesarean section due to fetal distress and maternal polyhydramnios.

### 2.3 Maternal history

The mother was 23 years old and had a thin, bony face, atrophied temples and masseter muscles like her sister (N1's mother). A positive history of limping and hand locking since the 16–17 year‐old was reported. She spoke loosely and her education in mathematics failed.

## 3 METHODS

The results of the sepsis workup were negative, the umbilical cord arterial blood gas (ABG) was normal, and brain ultrasonography in N1 was normal but in N2 showed 4 cystic structures in the left choroid plexus. On echocardiography, in N1, a large patent foramen ovale was detected and in N2, there was a patent ductus arteriosus. In the N1 X‐ray, the elevation of the right hemi‐diaphragm was observed (Figure 1).

Screening for metabolic diseases including ammonia, lactate, pyruvate, and ABG, was normal. Creatine phosphokinase (CPK) and lactate dehydrogenase (LDH) were in the normal range.

**FIGURE 3:**
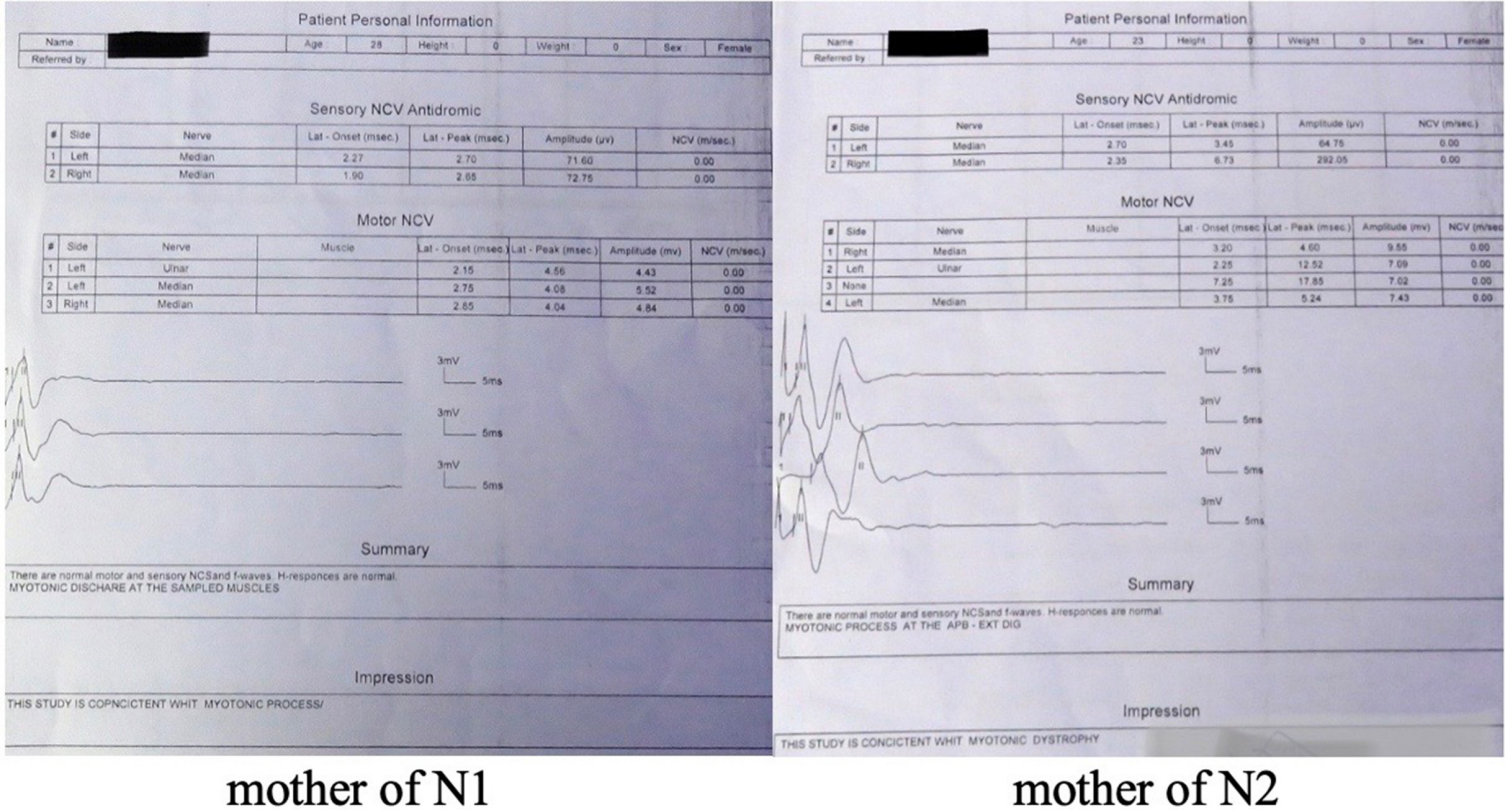
EMGs of mothers; Reveal myotonic discharge at the sampled muscles.

Myotonia in the form of hand locking was observed in both mothers during handshaking. Therefore, EMG‐NCV was performed for mothers which showed myotonic waves (Figure 3).

## 4 CONCLUSION AND RESULTS

Finally, the diagnosis of CDM was made based on characteristic findings on physical examination and electrophysiological studies of their mothers. We consulted an orthopedist for N1 clubfoot.

Treatment began for her with a serial casting session. Poor sucking was treated by a speech therapist, by manipulating the masticator muscles. The transition from PG feeding to breastfeeding was facilitated in N2, but N1 was discharged with PG feeding and her condition improved over 10 days and she was finally able to tolerate oral feeding.

During the patient's follow‐up, after 9 months, N1 sits briefly with the assistant and rolled around on the bed. She has no respiratory or feeding problems. At the age of 2 years old, the child sits easily, and stands with the help of household furniture, but does not walk or stand without help, and is currently undergoing occupational therapy. So far, he has not needed hospitalization due to respiratory problems, including pneumonia. She can only say six words.

After 9 months, N2 sits up alone without support. She creeps and crawls sometimes. She has no respiratory or feeding problems. At the age of 2 years old, She sits easily, walks, but does not say a word.

As a conclusion, neonatal hypotonia in first cousins after birth to a mother with polyhydramnios and a positive history of myotonia should be considered CDM until proven otherwise.

## 5 DISCUSSION

CDM is an autosomal dominant trait associated with increased CTG repeats in unstable DNA regions and age of onset, disease severity, and rate of progression correlated with the number of repetitions. The more CTG is repeated, the earlier the disease begins and the more severe it is. However, the diagnosis was finalized by a genetic study and CTG repeat counts^10^, ^11^, ^12^ but was supported especially by showing the myotonic discharges on EMG.

Polyhydramnios is one of the most common prenatal manifestations of many neuromuscular diseases, including CDM, and is caused by swallowing dysfunction.^13^ Several studies have reported varying rates of polyhydramnios in these patients. Zaki et al. reported polyhydramnios to occur in 82% of cases^14^ and this complication also occurred in 47% of cases in Zapata et al. Cohort study.^15^ In addition, decreased fetal movements and fetal distress are also other manifestations of the disease.^15^ Both of our babies have polyhydramnios, the first baby has decreased fetal movements and the second baby has fetal distress.

Respiratory problems are the main problem of CDM.^16^ In a cohort study, Zapata et al. stated that the most common long‐term morbidity is respiratory infections.^15^ However, this can occur due to lack of coordination when swallowing or weakness of the intercostal muscles or diaphragm, which can lead to aspiration. Our patient had few respiratory problems and feeding difficulty and swallowing disorder in the first days of life, but did not develop pneumonia during follow‐up care. The reason for this is thought to be that the breathing and swallowing disorder heals quickly.

Although the definitive diagnosis of this disease is by showing CTG repeat counts through genetic tests,^17^ However, these tests were not performed on these babies because in our country these tests are very expensive and have to be sent abroad, and the parents did not consent to the conduct of these tests. Based on the symptoms of the disease and the mother's EMG‐NCV results, the diagnosis of this disease in infants seems clear. Additionally, we perform this test on the mothers and not the newborn because this test is generally considered normal in newborns.^18^

A limitation of our report is the lack of availability of genetic studies, but neonatal hypotonia and other manifestation including hyporeflexia, respiratory problems, feeding difficulty, club‐foot, frog‐like posture, tent‐like opened mouth, right hemidiaphragm elevation in x‐ray graphy, … plus maternal polyhydramnios and myotonia (hand locking) may be sufficient for diagnosis.

The interesting and educational points in this presentation were the timing of their birth and the timing of their contracting this disease and the diagnosis of the disease in their mothers after their birth. A mother who had more serious problems had a sicker baby, and a mother who had fewer disorders, her baby had fewer problems and had a faster recovery and motor development.

## AUTHOR CONTRIBUTIONS

**Mousa Ahmadpour‐kacho:** Writing – review and editing. **Yadollah Zahed Pasha:** Writing – review and editing. **Samira Pournajaf:** Conceptualization; project administration; writing – review and editing.

## FUNDING INFORMATION

None.

## CONFLICT OF INTEREST STATEMENT

None declared.

## CONSENT

Written informed consent was obtained from the patient to publish this report in accordance with the journal's patient consent policy.

## Data Availability

None.
